# Methodology for clinical genotyping of *CYP2D6* and *CYP2C19*

**DOI:** 10.1038/s41398-021-01717-9

**Published:** 2021-11-22

**Authors:** Beatriz Carvalho Henriques, Avery Buchner, Xiuying Hu, Yabing Wang, Vasyl Yavorskyy, Keanna Wallace, Rachael Dong, Kristina Martens, Michael S. Carr, Bahareh Behroozi Asl, Joshua Hague, Sudhakar Sivapalan, Wolfgang Maier, Mojca Z. Dernovsek, Neven Henigsberg, Joanna Hauser, Daniel Souery, Annamaria Cattaneo, Ole Mors, Marcella Rietschel, Gerald Pfeffer, Stacey Hume, Katherine J. Aitchison

**Affiliations:** 1 Department of Psychiatry, University of Alberta, Edmonton, Canada; 2 Neuroscience and Mental Health Institute, University of Alberta, Edmonton, Canada; 3 Department of Biological Sciences, University of Alberta, Edmonton, Canada; 4 Department of Clinical Neurosciences, Cumming School of Medicine, Hotchkiss Brain Institute, University of Calgary, Calgary, Canada; 5 Department of Pharmacology, University of Alberta, Edmonton, Canada; 6 Department of Medical Genetics, University of Alberta, Edmonton, Canada; 7 Department of Psychiatry and Psychotherapy, University of Bonn, Bonn, Germany; 8 Institute Karakter, Ljubljana, Slovenia; 9 Croatian Institute for Brain Research, Centre for Excellence for Basic, Clinical and Translational Research, University of Zagreb School of Medicine, Zagreb, Croatia; 10 Departnent of Psychiatry, Poznan University of Medical Sciences, Poznań, Poland; 11 Laboratoire de Psychologie Médicale, Université Libre de Bruxelles and Psy Pluriel, Centre Européen de Psychologie Médicale, Brussels, Belgium; 12 Biological Psychiatry Unit, IRCCS Istituto Centro San Giovanni di Dio Fatebenefratelli, Brescia, Italy; 13 Department of Pharmacological and Biomolecular Sciences, University of Milan, via Balzaretti 9, 20133 Milan, Italy; 14 Psychosis Research Unit, Aarhus University Hospital, Risskov, Denmark; 15 Department of Genetic Epidemiology in Psychiatry, Central Institute of Mental Health, Medical Faculty of Mannheim, Heidelberg University, Mannheim, Germany; 16 Alberta Child Health Research Institute & Department of Medical Genetics, Cumming School of Medicine, University of Calgary, Calgary, Canada; 17 Alberta Precision Laboratories, Edmonton, Canada; 18 Alberta Health Services, Edmonton, Canada; 19 King’s College London, London, UK

**Keywords:** Clinical genetics, Pharmacogenomics

## Abstract

Many antidepressants, atomoxetine, and several antipsychotics are metabolized by the cytochrome P450 enzymes CYP2D6 and CYP2C19, and guidelines for prescribers based on genetic variants exist. Although some laboratories offer such testing, there is no consensus regarding validated methodology for clinical genotyping of *CYP2D6* and *CYP2C19*. The aim of this paper was to cross-validate multiple technologies for genotyping *CYP2D6* and *CYP2C19* against each other, and to contribute to feasibility for clinical implementation by providing an enhanced range of assay options, customizable automated translation of data into haplotypes, and a workflow algorithm. AmpliChip CYP450 and some TaqMan single nucleotide variant (SNV) and copy number variant (CNV) data in the Genome-based therapeutic drugs for depression (GENDEP) study were used to select 95 samples (out of 853) to represent as broad a range of *CYP2D6* and *CYP2C19* genotypes as possible. These 95 included a larger range of *CYP2D6* hybrid configurations than have previously been reported using inter-technology data. Genotyping techniques employed were: further TaqMan CNV and SNV assays, xTAGv3 Luminex *CYP2D6* and *CYP2C19*, PharmacoScan, the Ion AmpliSeq Pharmacogenomics Panel, and, for samples with *CYP2D6* hybrid configurations, long-range polymerase chain reactions (L-PCRs) with Sanger sequencing and Luminex. Agena MassARRAY was also used for *CYP2C19*. This study has led to the development of a broader range of TaqMan SNV assays, haplotype phasing methodology with TaqMan adaptable for other technologies, a multiplex genotyping method for efficient identification of some hybrid haplotypes, a customizable automated translation of SNV and CNV data into haplotypes, and a clinical workflow algorithm.

## Introduction

Many antidepressants, atomoxetine, and several antipsychotics are metabolized by CYP2D6 and CYP2C19 [1–7]. The gene (*CYP2D6*) encoding the enzyme CYP2D6 is on chromosome 22q13.2 [8] adjacent to two pseudogenes, *CYP2D7* and *CYP2D8* [9, 10]. The high homology between *CYP2D6* and these pseudogenes and the presence of flanking transposable genetic elements [11] makes the region vulnerable to the generation of variable copy numbers of the *CYP2D6* gene and hybrid genes made up of sequence derived in part from *CYP2D7* and in part from *CYP2D6* [12–21]. Such variants are challenging to characterize for many technologies. The *CYP2C19* gene encoding the CYP2C19 enzyme is located at chromosome 10q23.33, also together with other similar genes [22–25]. While structural variants of *CYP2C19* have recently been identified [26], the more commonly studied haplotypes result from single nucleotide variants (SNVs) [27]. Haplotypes in both genes are referred to as “star alleles,” e.g., *2, *3, etc. as defined by PharmVar [13], a consortium which maintains a curated catalog of allelic variation in genes impacting drug metabolism, disposition, and response.

Different *CYP2D6* or *CYP2C19* haplotypes may be associated with different levels of enzyme activity, ranging from loss-of-function haplotypes (which give rise to no functional enzyme), to haplotypes with decreased function (which are associated with an enzyme with reduced metabolic activity), to gain-of-function haplotypes (associated with increased activity) [4]. Haplotype frequencies vary between and within ethnic groups [1, 4, 28–31]. The study of clinical associations between variants in these genes and response to relevant medications has been to date limited by the challenging nature of the genotyping, particularly in the case of *CYP2D6* [32]. This gene is extremely polymorphic, with single or short sequence variants including indels (insertions/deletions), sequence derived from *CYP2D7* (described as “conversions” such as an exon 9 conversion), and structural variants (deletions of the entire *CYP2D6* gene, gene duplications/multiplications denoted as *xN*s, and hybrids [21] as above described).

There are now a number of laboratories offering testing for these genes for clinical utility; however, to date there is no consensus regarding validated methodology suitable for this purpose [33]. Clinical validation requires selecting appropriate haplotypes for testing, obtaining reference samples, and establishing test analytical validity and feasibility [34]. A recent paper provides recommendations on *CYP2D6* haplotype selection for clinical testing [35], and the Genetic Testing Reference Material Program (GeT-RM) has conducted extensive work in order to provide reference samples [36–38]. The aims of this paper were to cross-validate multiple technologies against each other for genotyping *CYP2D6* and *CYP2C19* thereby facilitating feasible clinical implementation through the provision of a range of assay options, to develop customizable and automated translation of data into haplotypes, and to recommend an efficient clinical workflow algorithm that includes hybrid configurations. CYP2D6 metabolizes many other medications in addition to psychotropics (in total ~20–25% of clinically used drugs [39–41]); this work is also relevant for these medications, many of which are prescribed as comedications in patients with psychiatric disorders.

In the GeT-RM publications, data are available from the AmpliChip CYP450 Test, the Luminex *CYP2D6* xTAG v3, and other genotyping platforms including AutoGenomics INFINITI, ParagonDx, and LDT SNaPShot, PharmacoScan, Agena MassARRAY iPLEX CYP2D6 v1.1, TaqMan assays, L-PCR, digital droplet PCR, and amplicon sequencing using next-generation sequencing (NGS) or long-read single-molecule real-time sequencing (SMRT, *N* = 3) [36–38]. *CYP2D6* TaqMan assays have been compared with data arising from mPCR-RETINA, Sanger sequencing, long-PCR for CYP2D6*5, and NGS data available via the 1000 Genomes Project [42]. Validation data have been provided for the Agena VeriDose Core and CYP2D6 copy number variation (CNV) Panel versus a proprietary panel and two TaqMan assays [43]. There are two technologies that provide SMRT sequencing: Pacific Biosciences and Oxford Nanopore; the former has been compared to data for *CYP2D6* from the AmpliChip CYP450 Test (which included 32 *CYP2D6* variant haplotypes including some structural variants but no hybrids) on 25 individuals in one study [44], and to data from targeted Illumina NGS in 17 individuals including one hybrid haplotype (CYP2D6*36) [45]; the latter has been used on 7 reference and 25 clinical samples (which included some structural variants but no hybrids) [46]. The former was also applied to 561 patients with breast cancer, and to replication samples, although with limitations, including pertaining to hybrid haplotypes [47]. While software exists to call *CYP2D6* haplotypes from next generation full sequencing data [48–51], such tools are not yet available for SMRT data, or for combinations of CNV and SNV data that arise from other technologies including NGS.

The novel contributions described herein include: (1) inter-technology concordance data on genotypes from genomic samples including a range of *CYP2D6* hybrids and hybrid tandems for the AmpliChip CYP450 test, TaqMan CNV and SNV assays, the Luminex *CYP2D6* and *CY2C19* xTAG v3 assays, the NGS AmpliSeq Pharmacogenomics Panel, PharmacoScan, and the Agena MassARRAY (for *CYP2C19*); (2) details of *CYP2D6* amplicon Sanger sequencing methodology including primers; (3) adaptation of the Luminex *CYP2D6* assay for amplicon sequencing and provision of concordance data for this versus Sanger sequencing so that other multiplex genotyping methods can also be adapted for efficient identification of hybrid haplotypes; (4) haplotype derivation files for the interpretation of combinations of *CYP2D6* CNV and SNV data including hybrids and *CYP2C19*; (5) development of a broader range of TaqMan SNV assays; (6) outline of haplotype phasing methodology with TaqMan adaptable for other technologies; and (7) a clinical workflow algorithm that includes hybrid configurations.

## Materials and methods

Ninety-five DNA samples (originating from venous blood) were selected from those previously genotyped for *CYP2D6* and *CYP2C19* using the AmpliChip CYP450 Test (Roche Molecular Systems, Pleasanton, USA) supplemented by the TaqMan assay C_469857_10 for CYP2C19*17 as part of the Genome-based therapeutic drugs for depression (GENDEP) study [52]. Participants were all of self-reported White European ancestry. GENDEP was originally approved by ethics boards at all participating centers and approval for the work described herein was also provided by the University of Alberta Health Research Ethics Board—Biomedical Panel. Written informed consent was provided by all participants. The AmpliChip identified 32 *CYP2D6* variant haplotypes (*2, *3, *4, *5, *6, *7, *8, *9, *10, *11, *14, *15, *17, *19, *20, *25, *26, *29, *30, *31, *35, *36, *40, *41, *114 (reported as *14A) *1×N (*×N* referring to more than one copy), *2×N, *4×N, *10×N, *17×N, *35×N, *41×N). In addition, it covered *CYP2C19* haplotypes *2 and *3. Sample DNA concentrations were ascertained using fluorimetry-based methods (Qubit or Quantifluor).

### TaqMan copy number variant (CNV) assays for *CYP2D6*

TaqMan CNV assays for *CYP2D6* (assay IDs: Hs04083572_cn and Hs00010001_cn for intron 2 and exon 9 respectively; Thermo Fisher Scientific) were run according to the manufacturer’s protocol on a ViiA7 Real-Time PCR System (Thermo Fisher Scientific). Assays were run in quadruplicate [53]. Data were analyzed using CopyCaller software version 2.1 (Thermo Fisher Scientific) with internal calibrators of known *CYP2D6* copy number according to the manufacturer’s instructions (using a confidence level of at least 95%, most being above 99%).

Samples for which the TaqMan CNV call across the two probes were not equal and hence indicative of *CYP2D6* hybrids were analyzed with a third probe (assay ID Hs04502391_cn for *CYP2D6* intron 6). These samples, and those representing as broad a range of *CYP2D6* and *CYP2C19* genotypes as possible by the AmpliChip CYP450 and TaqMan CYP2C19*17 assays, or “no call” for *CYP2D6* on the AmpliChip CYP450 were then taken forward for further analysis (*N* = 95). The 95 were thus enriched for complex structural variants and other difficult to detect genotypes. The following genotyping techniques were employed: Luminex *CYP2D6* xTAG v3 and Luminex *CYP2C19* xTAG v 3, PharmacoScan (Thermo Fisher Scientific, Waltham, MA, USA), Ion AmpliSeq Pharmacogenomics Panel (Thermo Fisher Scientific), and TaqMan Drug Metabolism Genotyping Assays (Thermo Fisher Scientific). Data arising from these were then used to select samples for the generation of amplicons by long-range polymerase chain reaction (known as L-PCR) [18, 20, 54–56].

### TaqMan SNV assays

Haplotype phasing for samples with three copies of the *CYP2D6* gene according to the TaqMan, Pharmacoscan, and/or AmpliSeq CNV probe data, and heterozygous SNV data was conducted by the following methodologies: TaqMan assays for the relevant *CYP2D6* SNVs on genomic DNA, and/or L-PCR specific for *CYP2D6* duplicated genes followed by genotyping of the L-PCR product using relevant TaqMan SNV assays. The TaqMan SNV assays used were for CYP2D6*2, *3, *4, *6, *35 and *41 (with assay IDs C_27102425_10, C_32407232_50, C_27102431_D0, C_32407243_20, C_27102444_F0, and C_34816116_20 respectively). The TaqMan SNV assays for CYP2D6*2, *4, *10 (assay ID: C__11484460_40), and *35 were used on genomic DNA to conduct haplotype phasing for samples with CNV data consistent with three *CYP2D6* genes including a hybrid gene. In addition, a TaqMan assay (assay ID C__25986767_70) was used to cross-validate a CYP2C19*2-defining SNV, rs4244285. Samples were run in duplicate on a ViiA7 Real-Time PCR System (Thermo Fisher Scientific), with genotype calling after visual inspection, outlier exclusion, and manual adjustment of C_T_ threshold settings as necessary. Data arising from duplicates were compared with each other using an automated method available from the authors at request.

### Luminex

The Luminex xTAG *CYP2D6* and *CYP2C19* Kits v3 (research use only versions) were run according to the manufacturer’s instructions using on a Luminex 200 system (Luminex Molecular Diagnostics, Inc., Toronto, ON, Canada). The assays use multiplex allele specific primer extension (ASPE) with a bead-based assay system. Haplotypes covered for *CYP2D6* are: CYP2D6*2–*12 (including the *5 gene deletion), *14, *15, *17, *29, *35, *41, and gene duplication. Multiple different *CYP2D6* haplotype translators are provided (10.6084/m9.figshare.16828741) owing to the Allele Typer software having a limit on the number of haplotypes that it can process in a given translator. These cover this range of haplotypes covered by the Luminex and also permit derivation of other haplotypes of known function (*20, *39.001, *39.002, *69, *114) and sub-haplotypes (*1.011, *2.001/*2.005/*2.012/*2.013/*2.018/*2.020/*2.021, *2.004, *4.002, *4.012, *6.003, *12.001, *12.002), hybrid haplotypes (*4.013, two specific *13s (EU093102 previously known as *66, and GQ162807 previously known as *77), *36, *57, *61, *63, *68, and *83) and hybrid tandems (*4.013 + *4, *4.013×2 + *4, *4.013 + *4 *×* *2*, *4.013 *×* *2* + *4 *×* *2*, *36 + *10, *36 + *10 *×* *2*, *36 *×* *2* + *10, *36 *×* *2* + *10 *×* *2*). We also provide other versions of the translators in which *70 or *107 replace a *39 haplotype. These latter versions are intended for research use. The RUO software for *CYP2C19* reports CYP2C19*2–*10, and *17. Our *CYP2C19* haplotype translator (10.6084/m9.figshare.16828738) also permits derivation of: *2.002/*2.010/*2.012, *4, *4.002, *5, *6, *7, *8, *9, and *10.

### Ion AmpliSeq pharmacogenomics panel

Genotyping using the Ion AmpliSeq Pharmacogenomics Panel (Thermo Fisher Scientific) was conducted according to the manufacturer’s instructions using an Ion Chef instrument (Thermo Fisher Scientific, Waltham, MA, USA). Short stretches of genomic DNA were sequenced, including regions of *CYP2D6* designed to detect *CYP2D6-*structural variants. Following sequencing, data were analyzed using the GeneStudio Data Analysis software (Thermo Fisher Scientific). Sequencing generated an average of 109,454 reads per sample (mean read length 142.5 bp), with two samples failing quality control (in a manner indicating likely insufficient template: mapped read numbers of 18 and 51). Variant calling by the Ion Torrent Variant Caller version 5.10.1.19 (Thermo Fisher Scientific) generated three text files: one with the genotype at each SNV (including 20 *CYP2D6* variants and 11 *CYP2C19* variants), one for the *CYP2D6* exon 9 CNV output, and one for the *CYP2D6* gene level CNV data (based on sequence across nine regions in *CYP2D6*) (10.6084/m9.figshare.16828747). Haplotype translation files were created (using data from PharmVar, hybrid haplotype data available on the archived Human Cytochrome P450 (CYP) Allele Nomenclature Committee page [57], and a relevant publication [17]) to derive *CYP2D6* and *CYP2C19* haplotypes including various hybrid configurations in conjunction with the AlleleTyper software (Thermo Fisher Scientific) [58].

### PharmacoScan

The PharmacoScan array-based technology was run at Neogen Genomics (Lincoln, NE, USA). The resultant data, including more than 100 variants in *CYP2D6* and 60 variants in *CYP2C19,* were analyzed using the Axiom Analysis Suite 4.0.3.3 (Thermo Fisher Scientific). Version r8 + 20200211 of the manufacturer’s *CYP2D6* haplotype translation file was used. This file was created using data from PharmVar, building on earlier work that used data available on the archived Human CYP Allele Nomenclature Committee page, and some contribution from the Aitchison laboratory. CNV calls were provided by probes for exon 9 of *CYP2D6* as well as for the 5′ and 3′ flanking regions as described [38].

### Long-range PCR assays with characterization of resultant amplicons

L-PCR was performed as described with minor modifications to generate an amplicon specific for the duplicated *CYP2D6* gene [55]. In brief, for the L-PCR assay that generates the D amplicon (specific for duplicated *CYP2D6* genes), we used primers as described [55], i.e., forward and reverse 5′-CCAGAAGGCTTTGCAGGCTTCAG-3′ and 5′-CGGCAGTGGTCAGCTAATGAC-3′, respectively, with minor modifications to the PCR conditions. Amplicons were purified by gel extraction (GeneJET Gel Extraction Kit, Thermo Fisher Scientific, Waltham, MA, USA), and genotyped using the TaqMan SNV assays described above.

Samples with unequal calls across the TaqMan, PharmacoScan, or AmpliSeq CNV probes were subjected to L-PCR assays to generate amplicons specific for *CYP2D6-2D7* or *CYP2D7-2D6* hybrids (E, G [20], or H [18]), with minor modifications. Amplicons were purified by gel extraction and subjected to Sanger sequencing (10 μl at 3.5 ng/μl per reaction) using BigDye Terminator version 3.1 chemistry, the Axygen CleanSEQ magnetic beads-based post-reaction clean up protocol (automated on a Biomek 3000 workstation), and a capillary 3130xl Genetic Analyzer (Thermo Fisher Scientific, Waltham, MA, USA).

Primers for sequencing (Supplementary Table 1) included novel ones designed for this study, those supplied by Dr. Gaedigk (personal communication), as well as previously reported ones used to generate the L-PCR amplicons and in prior literature [56, 59, 60]. Sequence traces were aligned (to sequences available via the PharmVar [13] or archived [57] *CYP2D6* pages) and analyzed using SnapGene software version 5.1.4.1 (GSL Biotech LLC, Chicago, IL, USA).

### The Agena MassARRAY

The Agena MassARRAY (Agena Bioscience, San Diego, CA, USA) uses matrix-assisted laser desorption/ionization-time of flight (MALDI-TOF) mass spectrometry technology for resolving oligonucleotides. We ran 8 *CYP2C19* variants to enable calling of 9 haplotypes. Genomic DNA was subjected to PCR followed by single-base extension with the extension products then being dispensed onto a SpectroCHIP Array and detected via mass spectrometry as described [38]. Haplotypes were assigned using Typer Analyzer software version v4.1.83 (Agena Bioscience).

## Results

### *CYP2C19*

Percentage concordance for Luminex, AmpliSeq, PharmacoScan, Agena, and prior data with consensus *CYP2C19* genotype are shown in Table 1. All technologies apart from the AmpliChip were able to detect CYP2C19*6 and *8. For CYP2C19*2 and CYP2C19*17, data from all technologies, where available, were concordant.

**Table 1:** Percentage concordance for Luminex, Ion S5, PharmacoScan, Agena, and prior data with consensus *CYP2C19* genotype.

Consensus genotype	*N*	Prior data: AmpliChip^a^ and TaqMan *17 (% concordance)	Luminex RUO (% concordance)	Ion S5 (% concordance)	PScan (% concordance)	Agena (% concordance)
*1/*1	44	100	100	97.7 (42/43)^b^	100 (42/42)^c^	95.2 (40/42)^d^
*1/*17	24	100	100	100 (22/22)	100 (23/23)	100 (22/22)
*1/*2	16	100	100	100	100	100
*17/*17	1	100	100	0(0/1)^e^	NA^f^	100
*2/*17	4	100	100	100	100	100 (3/3)
*2/*2	2	100	100 (1/1)	100^g^	100	100 (1/1)
*1/*8	1	0	100	100	100	100
*2/*6	1	0	100	100	100	100

For enhanced validation, two more samples of CYP2C19*17/*17 genotype by TaqMan were genotyped: one on IonS5, PharmacoScan and Luminex (concordant on all three technologies), and one on Luminex (concordant). A TaqMan assay for CYP2C19*2 cross-validated AmpliChip data 100% in the full GENDEP dataset.

^a^ Note that the overall concordance for the AmpliChip data without the CYP2C19*17 by TaqMan was 64/96 = 66.7%.

^b^ One “no call” out of the 43 genotyped using this assay.

^c^ A couple of CYP2C19*27 haplotypes were found in this group; this is now classified as CYP2C19*1.006.

^d^ Two “no calls” out of the 42 genotyped using this assay.

^e^ One “no call”.

^f^ Not assayed.

^g^ Two samples for which the specific options *2/*2.002, or *2/*2.010, or *2/*2.012.

### *CYP2D6*

Comparative genotypic and CNV data across the technologies for samples with one and three copies of the *CYP2D6* gene are shown in Supplementary Tables 2 and 3, respectively. Owing to the “no calls” in the AmpliSeq CNV data, we revised these to manual calls, where possible, after reviewing the vcf files. This did result in an improvement in the degree of concordance with consensus genotypes for the AmpliSeq. Data for samples with a CNV call of two are shown in Supplementary Table 4.

While the AmpliChip provided haplotype phasing of *CYP2D6xNs* (i.e., assignment of the duplication/multiplication to one or other of the two chromosomes), the other technologies included herein do not offer that. We used TaqMan assays on genomic DNA to identify which haplotype was duplicated/multiplicated based on relative magnitude of signals arising from TaqMan wild-type and mutant probes for each assay (Supplementary Fig. 1). All of our *CYP2D6xNs* were duplications with the exception of one sample, which had a CYP2D6*41×3. Consistent with this, our TaqMan CNV data were 4, 4, 4, and the raw PharmacoScan copy number probe calls were 4, 4, 4.

Data including consensus genotypes for the samples with hybrids genotyped to date are shown in Supplementary Table 5. Nineteen samples had an unequal call across at least two out of three CNV probesets (TaqMan, PharmacoScan, or AmpliSeq); for nine of these, the CNV pattern was consistent with a *CYP2D7-2D6* hybrid, and for 10 with a *CYP2D6–2D7* hybrid. For all of the *CYP2D6–2D7* hybrids, the pattern was consistent with an extra *CYP2D6* gene, either on the same haplotype as the hybrid gene (in cis), or on the other chromosome 22 (in trans). This was also the case for five of the *CYP2D7–2D6* hybrids. Amplicons consistent with hybrids [17–20] were generated for all 19 samples. Six samples had an unequal call across CNV probes for only one platform; four of these were genotyped as *CYP2D6* duplications (CYP2D6*1×2/*4, *1/*1×2, *2×2/*1 and *2×2/*35) and two as heterozygotes (*1/*2 and *1/*3.001). For three of these, amplicon G was generated; however, it should be noted that the primer pair for this amplicon will also amplify up *CYP2D7* (Fig. 2 in Black et al. (2012) [20], observed where the *CYP2D6* downstream gene was *1, *4, or *41; these three all had genotypes including the *1 and/or *4, specifically *1/*2, *1×2/*4, *4×2/*1).

L-PCR amplicons specific for *CYP2D7–2D6* hybrids aligned well to CYP2D6*13 sequences Specifically, three samples aligned to the GQ162807 sequence (previously CYP2D6*77) deposited by Gaedigk et al. (2010) [18], where CYP2D6*13 is found in tandem arrangement with CYP2D6*2. The consensus genotype (from data including TaqMan assays) for these samples was CYP2D6*13 + *2/*1. Three other amplicons aligned well to the EU093102 sequence for another CYP2D6*13 variant, which is found as a single gene on one chromosome [15, 16], consistent with the consensus genotypes for these samples: CYP2D6*13/*4.013, CYP2D6*13/*1, and CYP2D6*13/*1 (Supplementary Table 5). At this point, we have two remaining CYP2D6*13s for which the exact CYP2D6*13 has not been identified; however, we have sufficient data to make a consensus genotype call for both (CYP2D6*13 + *4/*5, and CYP2D6*13 + *2/*41) and all CYP2D6*13 haplotypes have the same CYP2D6 enzyme activity score (zero, i.e., do not encode any functional CYP2D6 protein) owing to a T insertion in exon 1 that is a frameshift mutation resulting in premature chain termination [21]. L-PCR amplicons for *CYP2D6-2D7* hybrids aligned to EU530605 (CYP2D6*4-like [17]), with one aligning to EU530606 (partial sequence for CYP2D6*68 [17]).

CYP2D6*13 + *4/*5 represents a novel haplotype. The CNV data from TaqMan and AmpliSeq were 1, 2, 2 and 1, 2 consistent with the presence of a *CYP2D7–2D6* hybrid (a CYP2D6*13) with a switch region between intron 2 and intron 6. On alignment of the Sanger-sequencing data, the sequence appeared as if the sample was heterozygous from a region consistent with this inferred switch region onwards, with the region prior to this aligning well to *CYP2D7*. This would be consistent with our long-PCR having amplified up both a *CYP2D7[REP6]* (a version of *CYP2D7* that has a *CYP2D6* version of the repeat element at its 3′ region, consistent with the 3′ primer used for the long-PCR) and a CYP2D6*13 (legacy designations for the CYP2D6*13 haplotypes CYP2D6*67, *78, and *80 [1, 18, 19, 21, 57] have switch regions in the relevant area). Comparative data from other technologies for this sample showed genotypes of CYP2D6*4/*4 (AmpliChip), and no call with alternative calls of CYP2D6*4/UNK or *4.009/UNK (Pharmacoscan). These indicate that a CYP2D6*4 haplotype is also present. Haplotype phasing with the CYP2D6*4 TaqMan assay indicated a deletion on one allele and the CYP2D6*4 on the other. Therefore, we deduced a configuration of CYP2D6*13 + CYP2D6*4/CYP2D6*5, where the CYP2D6*5 deletion has a *CYP2D7[REP6]* followed by a deletion of the *CYP2D6* gene.

We also subjected the L-PCR amplicons to genotyping using the Luminex *CYP2D6* assay, with a protocol modification. The resultant genotypes at 14 SNVs were consistent with the sequence data (Table 2A, B). For example, the 4181G>C (rs1135840) variant was found in the Luminex and sequence data for samples aligned to GQ162807 or EU093102, while the 2851C>T (rs16947) variant was wild-type for samples aligned to EU093102 and variant for samples aligned to GQ162807. For the *CYP2D6–2D7* hybrids, our *CYP2D6* haplotype translator was able to identify hybrids including hybrid tandems (Supplementary Table 5).

**Table 2:** Comparative Sanger sequencing and Luminex genotyping data for *CYP2D6* variants in samples with (A) *CYP2D7–2D6* hybrid haplotypes and (B) *CYP2D6–2D7* hybrid haplotypes.

(A)															
Sample	Method	31G>A	100C>T	124G>A	137_138insT	882G>C	1660G>A	1708delT	1847G>A	2550delA	2851C>T	2936A>C	2989G>A	3184G>A	4181G>C
EU093102	NA	MUT	WT	WT	MUT	WT	WT	WT	WT	WT	WT	WT	Sequence not present	WT	MUT
GQ162807	NA	MUT	WT	WT	MUT	WT	WT	WT	WT	WT	MUT	WT	WT	WT	MUT
1	Luminex	MUT	WT	WT	MUT	WT	WT	WT	WT	WT	WT	WT	Low Signal	WT	MUT
	Sanger	MUT	WT	WT	MUT	WT	WT	WT	WT	WT	WT	WT	Sequence not present	WT	MUT
3	Luminex	MUT	WT	WT	MUT	WT	WT	WT	WT	WT	MUT	WT	WT	WT	MUT
	Sanger	MUT	WT	WT	MUT	WT	WT	WT	WT	WT	MUT	WT	WT	WT	MUT

(B)																
Method	−1584C>G	31G>A	100C>T	124G>A	138insT	883G>C	1023C>T	1659G>A	1707delT	1758G>T/A	1758G>T/A	1846G>A	2549delA	2850C>T	2988G>A	3183G>A
Luminex	WT	WT	MUT	WT	MUT	WT	WT	WT	WT	WT	WT	MUT	WT	WT	WT	WT
Sanger	WT	WT	MUT	WT	MUT	WT	WT	WT	WT	WT	WT	MUT	WT	WT	WT	WT

Sample 1 aligns to EU093102, sequence for the CYP2D6*13 haplotype previously known as CYP2D6*66.

Sample 3 aligns to GQ162807, sequence for the CYP2D6*13 haplotype previously known as CYP2D6*77 and found in a tandem arrangement with CYP2D6*2.

Sample aligns to EU530605 (a CYP2D6*4.013 hybrid haplotype).

## Discussion

**Fig. 1:**
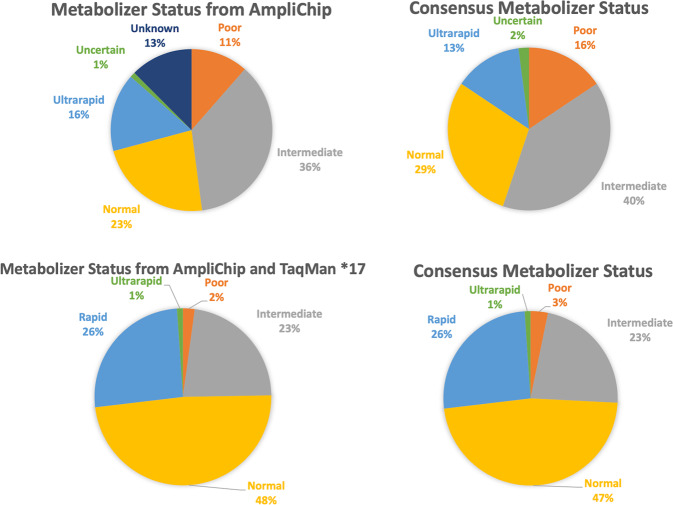
Revision in Deduced Metabolizer Status for *CYP2D6* and *CYP2C19*. **A** Change in distribution of *CYP2D6* metabolizer status deduced from genotype, from prior data to revised consensus data. **B** Change in distribution of *CYP2C19* metabolizer status deduced from genotype, from prior data to revised consensus data.

Consensus genotypes generated in 95 samples for *CYP2D6* and 93 samples for *CYP2C19* to date resulted in revision of assigned enzyme activity score for 28/95 (29%) and 2/93 samples (2.2%) for CYP2D6 and CYP2C19, respectively (sample selection enriched for structural variants in *CYP2D6*). These changes in assigned activity score were due to both changed genotype assignments and to new genotype assignments for samples that were “no calls” on AmpliChip (Fig. 1). For *CYP2C19*, the highest concordance with consensus genotype was in the Luminex and PharmacoScan data (100%). Data from Luminex, Agena, TaqMan, AmpliSeq, and PharmacoScan were 100% concordant for the CYP2C19*2 and CYP2C19*17, the most common loss-of-function and gain-of-function haplotypes, respectively, in individuals of European ancestry. No adjustments in the prior AmpliChip and TaqMan data were therefore required for either of these haplotypes; prior clinical association analyses conducted on the basis of these *CYP2C19* haplotypes are therefore valid [52, 61].

For *CYP2D6*, all technologies other than the AmpliChip were able to reliably detect the CYP2D6*5. Haplotype phasing of *CYP2D6xNs* was achieved by using relevant TaqMan assays on genomic DNA (Supplementary Fig. 1), or by genotyping an amplicon specific for the *xN*. Although using allelic ratios to cluster TaqMan genotype data leaves a degree of uncertainty around genotypes (e.g., if only one probe amplifies, it may not be possible to distinguish between C/C, CC/C, CC/− and C/−), this technique can be used effectively to distinguish different heterozygote groups (Supplementary Fig. 1). A strength of the sample set was the availability of prior AmpliChip data including haplotype phasing of *CYP2D6xNs*. The haplotype phasing achieved with our TaqMan allelic ratio method was consistent with the prior data, where available. One sample was genotyped as having a multiplication (i.e., more copies than 2), specifically of CYP2D6*41, which has been previously described [55, 62]. The majority of the revisions in assigned enzyme activity score were due to the inability of AmpliChip to detect hybrids (Supplementary Table 5) and a sensitivity issue for CYP2D6*5 detection by AmpliChip. The latter has been previously reported [44, 53] by us and other investigators, and relates to the particular sequence used to design detection of CYP2D6*5 by the AmpliChip.

**Fig. 2:**
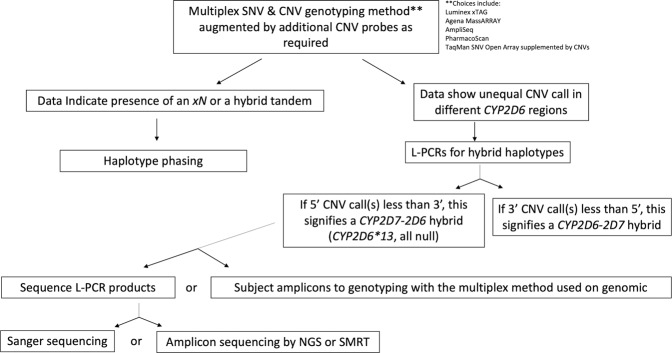
Clinical workflow algorithm for *CYP2D6* genotyping.

A focus of recent research on *CYP2D6* is the hybrid haplotypes [17–20]. We have developed efficient methodology for characterizing a range of hybrid haplotypes: a haplotype translation tool for the interpretation of combinations of *CYP2D6* CNV and SNV data including some hybrids and hybrid tandems (Supplementary Table 1), methodology for *CYP2D6* Sanger sequencing, and adaptation of the Luminex *CYP2D6* assay for amplicon sequencing with provision of concordance data for this versus Sanger sequencing to facilitate the application of other multiplex technologies to hybrid amplicons. In addition, we have developed a custom assay (ANT2NCE) that works on L-PCR amplicons for the sequence that occurs in *CYP2D6* hybrids such as CYP2D6*36 representing a *CYP2D7* exon 9 conversion. We also provide an algorithm for efficient clinical workflow that includes hybrid haplotypes including hybrid tandems (Fig. 2). The high degree of concordance between amplicon Luminex *CYP2D6* and Sanger sequencing data is important and extends the coverage of the Luminex *CYP2D6* assay to hybrids. Unfortunately, since we made this discovery, the assay has been withdrawn from the market. However, its components are still available for use as a laboratory developed test. Other multiplex assays currently available (such as the AmpliSeq Pharmacogenomics Panel, and the Agena MassARRAY Veridose Core plus CNV) could be likewise adapted. Of note, the workflow algorithm is capable of being adapted for *CYP2C19*, for which structural variants have been recently identified.

The efficient clinical workflow algorithm for *CYP2D6* genotyping provided in Fig. 2 includes: multiplex SNV and CNV assays, haplotype phasing, and L-PCRs with multiplex genotyping or sequencing (Fig. 2). Appropriate positive controls (e.g., from the GeT-RM [63], especially for the haplotypes that we did not see in this European sample set and which might be found in other ethnic groups, should be run with the assays. We have provided cross-validation data between a multiplex assay and Sanger sequencing for amplicons from CYP2D6*4.013 haplotype and CYP2D6*13 haplotypes (Table 2A, B). These can act as reference data for other multiplex assays processing hybrid amplicons. Laboratories desiring to use this workflow could use one of the technologies offering multiplex SNV and CNV data (e.g., the Open Array could be supplemented by TaqMan CNV assays) on genomic DNA, with the resultant data suggesting samples with hybrid alleles; these samples would require L-PCR amplicon generation and subsequent testing using the same multiplex assay as used on the genomic DNA (adapted as necessary). Factors influencing choice of technology by a particular laboratory may include: its local availability, relevant expertize, cost, minimum and maximum number of samples per run, any required data interpretation including bioinformatics, and turnaround time. Laboratories needing to process a small number of samples rapidly (e.g., for clinicians in acute care settings) could select an assay with a low minimum number of samples per run (e.g., Luminex). Others needing to process a large number of samples rapidly could choose a large maximum number of samples per run, such as NGS or array options. The technologies have other strengths and weaknesses, e.g., in regard to their coverage of *CYP2D6* CNVs. The Ion AmpliSeq Pharmacogenomics Panel provides the greatest number of regions that may be used for *CYP2D6* CNV derivation; PharmacoScan provides 5′ and 3′ UTR CNV probes; and TaqMan provides CNV assays for intron 2, intron 6, and exon 9. A TaqMan CNV assay for the 5′ UTR is also available (Hs07545275_cn) [45].

To increase the breadth of *CYP2D6* coverage to haplotypes in Tiers 1 and 2 of the recommendations by Pratt et al. we developed custom TaqMan assays: ANCFHM6 for rs61736512, which is part of the defining variant in CYP2D6*29, and ANWRUE2 for rs72549356 in CYP2D6*40. In regard to other haplotypes listed herein, although CYP2D6*39 is of normal function [64, 65], the enzyme activity for individuals of this haplotype in diverse ancestral populations is as yet unknown. Therefore, arguably, it can be justified to cover this haplotype in clinical genotyping. One of our samples, with a consensus *CYP2D6* genotype of *10/*41 had an alternative genotype of CYP2D6*39.001/*69. The list of haplotypes covered by assays reported herein also includes haplotypes of known function but to date without reference samples (CYP2D6*12 and CYP2D6*69) [35, 66–68]. Should these be identified in clinical genotyping and confirmed using a second method, then reference samples could be made available to other research and clinical labs. The rationale for inclusion of CYP2D6*70 ([69]; rated as uncertain in function, with a moderate evidence level by PharmVar [13]) and CYP2D6*107 ([70]; rated as unknown function, limited evidence level [13]) is less strong. We suggest including them on a research basis to maximize potential utility gaining knowledge relevant to clinical testing in diverse populations. When we used a *CYP2D6* translator with the *107 included, this resulted in several alternative calls in which a *1 was substituted by a *107.

In regard to *CYP2C19*, the recommendation of Pratt et al. (2018) [71] covers *CYP2C19* change-of-function haplotypes of ≥1% in any ethnic group [72], which include CYP2C19*2-*10, *17, and *35). We also suggest an additional TaqMan assay (C_312628039_10) for the c.463G>T variant (rs374036992) that may be found on the CYP2C19*17 haplotype and introduces a premature stop codon [73], and an assay to enable CYP2C19*17 haplotype phasing [73].

We acknowledge several limitations of this work. Firstly, we have not covered rare variants. It has been estimated that ~6.3% of the variance in olanzapine concentration is accounted for by rare *CYP2D6* variants, while rare variants are estimated to account for 4.4% of the overall genetic variability of CYP2C19 function [74]. Such variants would be identifiable by using methods that we have not validated, such as SMRT. While there are some papers reporting the use of SMRT to identify *CYP2D6* variants [44, 45, 47], SMRT has however, not yet been validated on as broad a range of hybrid configurations as were included in the present report. Secondly, the work was conducted in a set of samples from European individuals being treated for depression, with samples being selected as being representative for genotypes available in the whole set and with enrichment for *CYP2D6* structural variants. As such, we did not find *CYP2D6* haplotypes that would be more commonly found in other ethnic groups, such as *29. Therefore although the technologies are theoretically able to identify this haplotype, our lack of detection prevented confirmation. Of note, there are reference samples for test validation available with this haplotype from the Genetic Testing Reference Material Program (GeT-RM) [63]. Thirdly, theoretically it is possible that our CNV detection methods resulted in false positive calls for copy number loss in introns 2 and 6, owing to sequence variation in the relevant regions [75]. However, as we used three different technologies (AmpliSeq, Pharmacoscan, and TaqMan), covering probes in multiple regions of *CYP2D6* in addition to introns 2 and 6, and subjected any putative hybrid haplotypes to L-PCR and Sanger sequencing, we do not think this is a significant concern.

In summary, this study provides cross-validation data on a range of *CYP2D6* and *CYP2C19* genotypes including *CYP2D6* hybrids and hybrid tandems for several assays including: AmpliChip CYP450, TaqMan CNV and SNV assays, xTAGv3 Luminex *CYP2D6* and *CYP2C19*, the Agena *CYP2C19* content from the Veridose Core, PharmacoScan, and the Ion AmpliSeq Pharmacogenomics Panel. In addition, we provide the first reference data for multiplex assay amplicon genotyping for some *CYP2D6* hybrid haplotypes using long-range polymerase chain reactions (L-PCRs) followed by Sanger sequencing and Luminex. We have also developed a broader range of TaqMan SNV assays, and haplotype phasing methodology with TaqMan that is adaptable for other technologies. Finally, we have established a multiplex genotyping method for efficient identification of some hybrid haplotypes and created a customizable automated translator of SNV and CNV data for haplotype assignment. Together this work has laid the foundation for an efficient clinical workflow algorithm.

## Supplementary information

Supplementary Figure 1

Supplementary Table 1

Supplementary Table 2

Supplementary Table 3

Supplementary Table 4

Supplementary Table 5
