# Correction: Methodology for clinical genotyping of *CYP2D6* and *CYP2C19*

**DOI:** 10.1038/s41398-022-01845-w

**Published:** 2022-03-08

**Authors:** Beatriz Carvalho Henriques, Avery Buchner, Xiuying Hu, Yabing Wang, Vasyl Yavorskyy, Keanna Wallace, Rachael Dong, Kristina Martens, Michael S. Carr, Bahareh Behroozi Asl, Joshua Hague, Sudhakar Sivapalan, Wolfgang Maier, Mojca Z. Dernovsek, Neven Henigsberg, Joanna Hauser, Daniel Souery, Annamaria Cattaneo, Ole Mors, Marcella Rietschel, Gerald Pfeffer, Stacey Hume, Katherine J. Aitchison

**Affiliations:** 1 Department of Psychiatry, University of Alberta, Edmonton, Canada; 2 Neuroscience and Mental Health Institute, University of Alberta, Edmonton, Canada; 3 Department of Biological Sciences, University of Alberta, Edmonton, Canada; 4 Department of Clinical Neurosciences, Cumming School of Medicine, Hotchkiss Brain Institute, University of Calgary, Calgary, Canada; 5 Department of Pharmacology, University of Alberta, Edmonton, Canada; 6 Department of Medical Genetics, University of Alberta, Edmonton, Canada; 7 Department of Psychiatry and Psychotherapy, University of Bonn, Bonn, Germany; 8 Institute Karakter, Ljubljana, Slovenia; 9 Croatian Institute for Brain Research, Centre for Excellence for Basic, Clinical and Translational Research, University of Zagreb School of Medicine, Zagreb, Croatia; 10 Department of Psychiatry, Poznan University of Medical Sciences, Poznań, Poland; 11 Laboratoire de Psychologie Médicale, Université Libre de Bruxelles and Psy Pluriel, Centre Européen de Psychologie Médicale, Brussels, Belgium; 12 Biological Psychiatry Unit, IRCCS Istituto Centro San Giovanni di Dio Fatebenefratelli, Brescia, Italy; 13 Department of Pharmacological and Biomolecular Sciences, University of Milan, via Balzaretti 9, 20133 Milan, Italy; 14 Psychosis Research Unit, Aarhus University Hospital, Risskov, Denmark; 15 Department of Genetic Epidemiology in Psychiatry, Central Institute of Mental Health, Medical Faculty of Mannheim, Heidelberg University, Mannheim, Germany; 16 Alberta Child Health Research Institute & Department of Medical Genetics, Cumming School of Medicine, University of Calgary, Calgary, Canada; 17 Alberta Precision Laboratories, Edmonton, Canada

**Keywords:** Clinical genetics, Pharmacogenomics

Correction to: *Translational Psychiatry* 10.1038/s41398-021-01717-9, published online 22 November 2021

The original version of this article unfortunately contained an error in the affiliations and in Table 1. The original article has been corrected.

