# Supplementary material for: Methodology for clinical genotyping of CYP2D6 and CYP2C19
Source: Transl Psychiatry. 2021 Nov 22;11:596. doi: 10.1038/s41398-021-01717-9 (PMC8608805; doi:10.1038/s41398-021-01717-9)
Supplement: Supplementary file 1 — Supplementary Figure 1 [file 41398_2021_1717_MOESM1_ESM.docx]

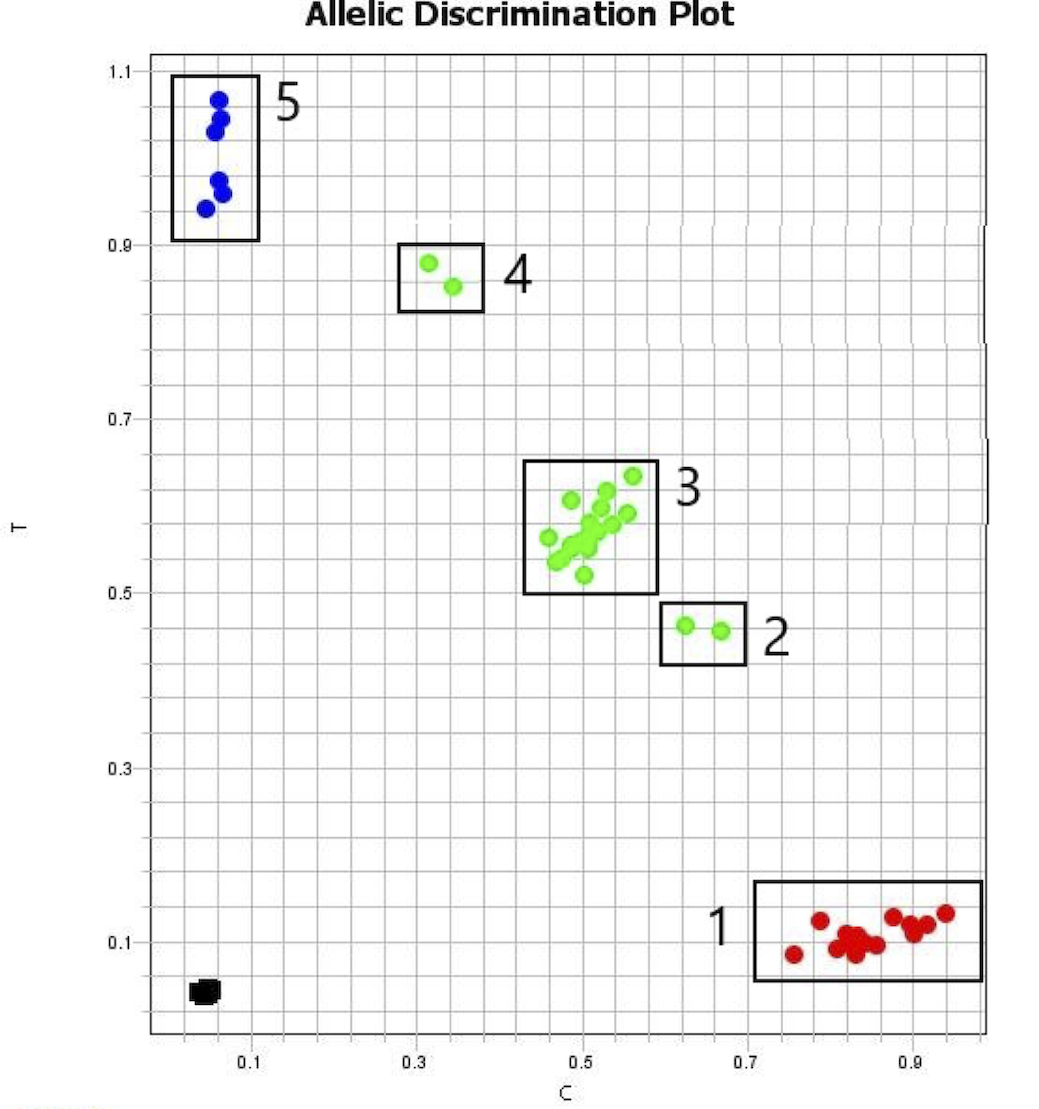


Supplementary Figure 1A-F. TaqMan *CYP2D6*41* allelic discrimination plot (A), with multicomponent plots (B-F) showing different allelic ratios corresponding to the clusters marked 1-5.  This is a C(Ref)>T(Var) SNV.


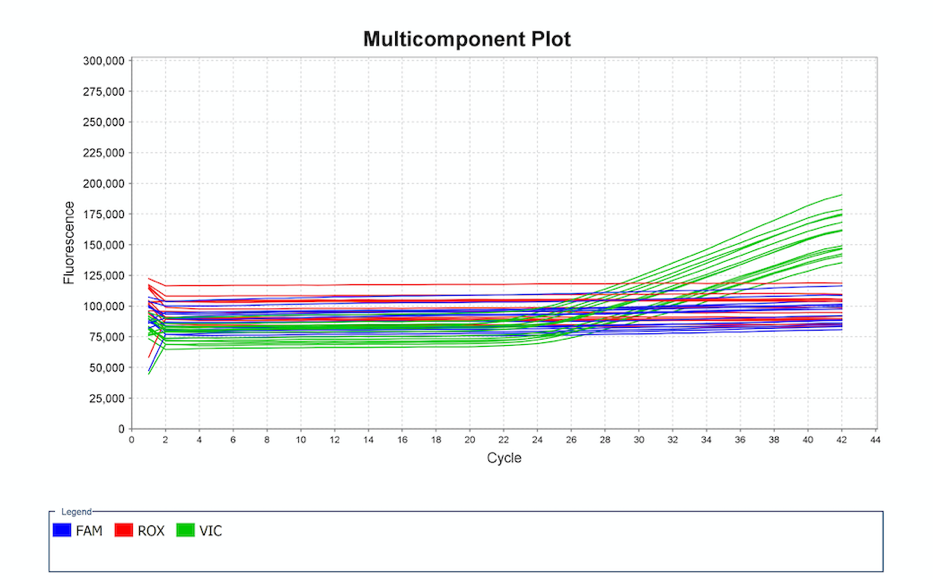


(B) Amplification of only C. Sample genotypes are **1/*1, *1/*1, *1XN/*1, *1X2/*2, *2X2/*1, *1X2/*5, *1X2/*35.*


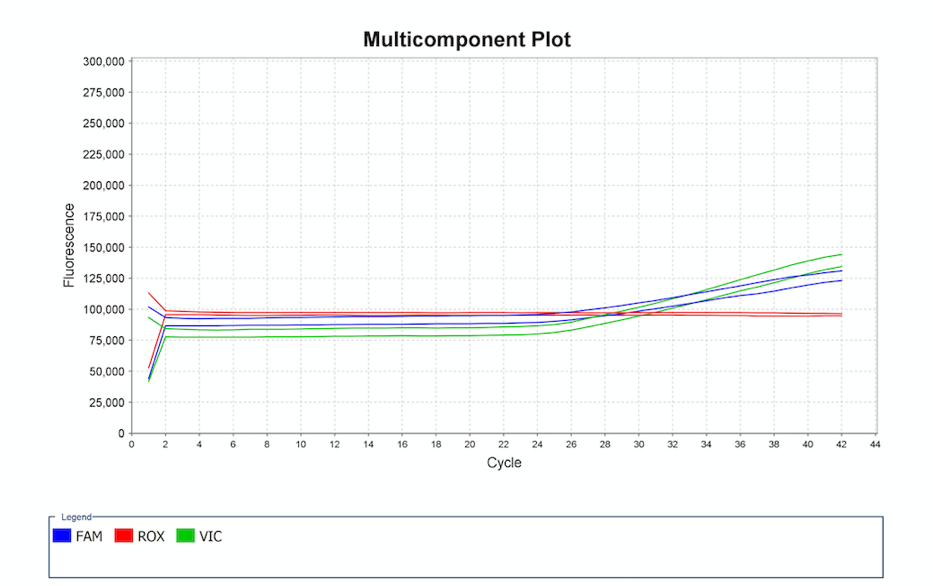
(C) Amplification of C and T, the former more than the latter, indicating a heterozygous **41* sample with more copies of C than T.  Sample genotype is **2X2/*41*.


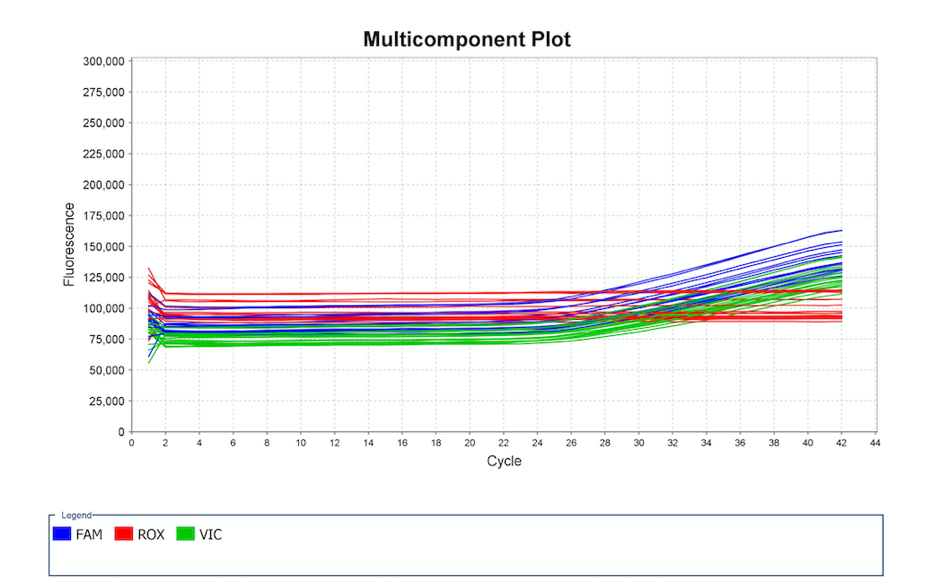


(D) Amplification of C and T to the same extent. Sample genotypes are **1/*41, *2/*41, *2/*41, *4/*41, *4/*41, *6/*41, *9/*41, *10/*41, *35/*41.*


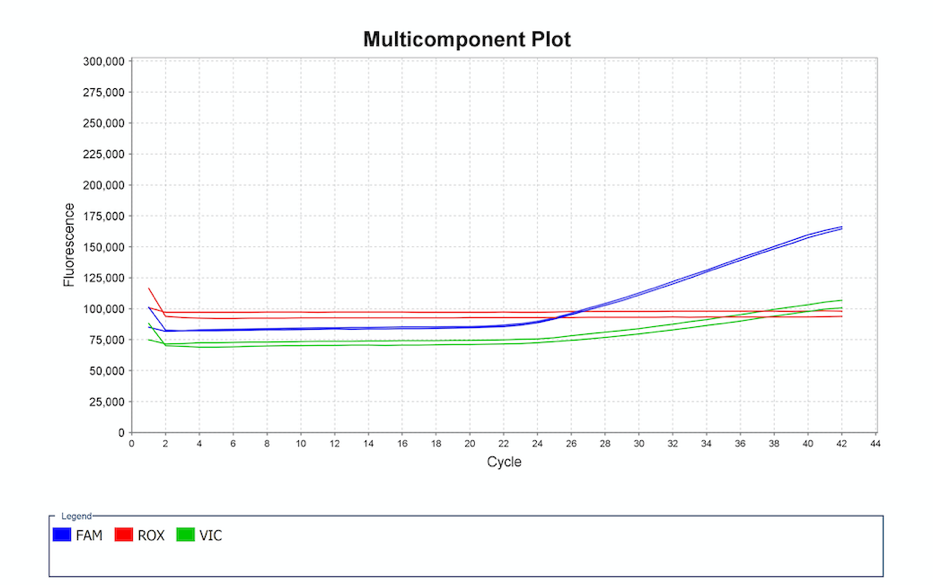


(E) Amplification of C and T, the latter more than the former. Sample genotype is **41X3/*3*.


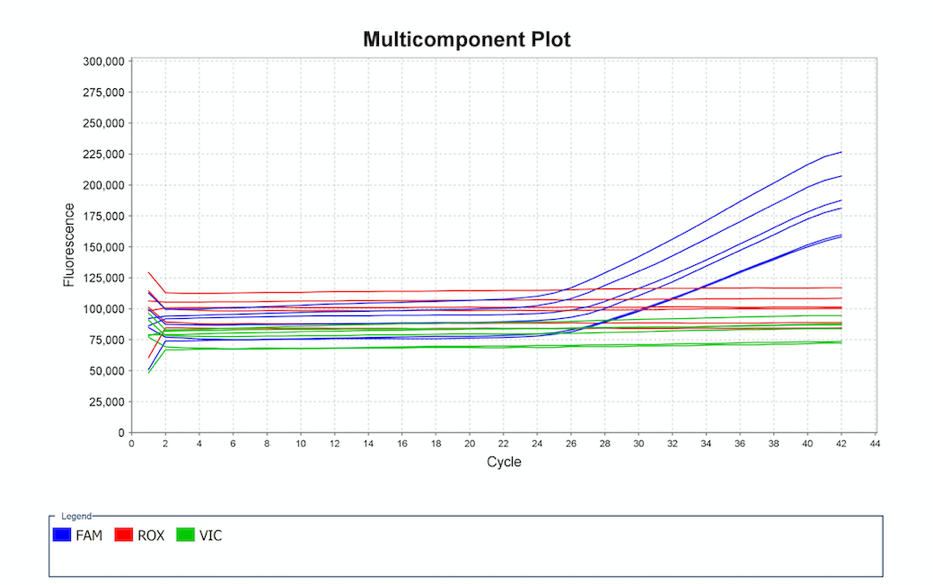
(F) Amplification of only T. Sample genotypes are **5/*41, *5/*41 *41/**41 (where **5* is a deletion of *CYP2D6*).
