# Supplementary material for: Methodology for clinical genotyping of CYP2D6 and CYP2C19
Source: Transl Psychiatry. 2021 Nov 22;11:596. doi: 10.1038/s41398-021-01717-9 (PMC8608805; doi:10.1038/s41398-021-01717-9)
Supplement: Supplementary file 3 — Supplementary Table 2 [file 41398_2021_1717_MOESM3_ESM.docx]

**Table S4. Comparative *CYP2D6* data for samples with copy number data indicating one copy of the gene, providing consensus genotype, and previous and new predicted enzyme phenotypes**

| **TaqMan CNV data** | | | **PScan CNV data** | | | **IonS5** | **IonS5** | **AmpliChip** | **Luminex (genomic) RUO** | **PScan** | **Ion S5** | **Consensus genotype** | **Activity Score Consensus** | **Previous Phenotype** | **New Phenotype** |
| --- | --- | --- | --- | --- | --- | --- | --- | --- | --- | --- | --- | --- | --- | --- | --- |
| **I2** | **I 6** | **E9** | **5’** | **E9** | **3’** | **gene** | **E9** |  |  |  |  |  |  |  |  |
| 1 | 1 | 1 | 1 | 1 | 1 | 1 | 1 | **4xN/*5* | **4/*5* | **4/*5* | **4/*5* | **4/*5* | 0 | PM | PM |
| 1 | 1 | 1 | 1 | 1 | 1 | 1 | 1 | **5/*35* | **5/*35* | **5/*35* | **5/*35* | **5/*35* | 1 | NM | IM |
| 1 | 1 | 1 | 1 | 1 | 1 | 1 | 1 | **2/*2* | **2/*5* | **2/*5* | **2.001, *2.005, *2.012, *2.013, *2.018, *2.020, *2.021/*5* | **2/*5* | 1 | NM | IM |
| 1 | 1 | 1 | 1 | 1 | 1 | 1 | 1 | **5/*10* | **5/*10* | **5/*10* | **5/*10* | **5/*10* | 0.25 | IM | IM |
| 1 | 1 | 1 | 1 | 1 | 1 | 1 | 1 | **1/*4* | **1/*5* | **1/*5* | **1/*5* | **1/*5* | 1 | NM | IM |
| 1 | 1 | 1 | 1 | 1 | 1 | 1 | 1 | **5/*41* | **5/*41* | **5/*41* | **5/*41* | **5/*41* | 0.5 | IM | IM |
| 1 | 1 | 1 | 1 | 1 | 1 | 1 | 1 | **1/*2xN* | **1/*5* | **1/*5* | **1/*5* | **1/*5* | 1 | UM | IM |
| 1 | 1 | 1 | 1 | 1 | 1 | 1 | 1 | **2/*4* | **2/*5* | **2/*5* | **2.001, *2.005, *2.012, *2.013, *2.018, *2.020, *2.021/*5* | **2/*5* | 1 | NM | IM |
| 1 | 1 | 1 | 1 | 1 | 1 | 1 | 1 | **3/*5* | **3/*5* | **3.001/*5* | **3/*5* | **3/*5* | 0 | PM | PM |
| 1 | 1 | 1 | 1 | 1 | 1 | 1 | 1 | **5/*35* | **5/*35* | **5/*35* | **5/*35* | **5/*35* | 1 | NM | IM |
| 1 | 1 | 1 | 1 | 1 | 1 | 1 | 1 | **4/*35* | **3/*5* | **3.001/*5* | **3/*5* | **3/*5* | 0 | NM | PM |
| 1 | 1 | 1 | 1 | 1 | 1 | 1 | 1 | **5/*41* | **5/*41* | **5/*41* | **5/*41* | **5/*41* | 0.5 | IM | IM |
| 1 | 1 | 1 |  |  |  |  |  | **4/*5* | **4/*5* |  | *NA* | **4/*5* | 0 |  |  |

PScan = PharmacoScan, I2 = intron 2, I6 = intron 6, E9 = exon 9, 5’ = 5’ flanking region, 3’ = 3’ flanking region

Bold font denotes adjustments in data owing to new genotypic data

Blue font denotes adjustments in data owing to revised phenotypic definitions based on activity score^1^

References

1. Caudle K.E., *et al*. Standardizing CYP2D6 Genotype to Phenotype Translation: Consensus Recommendations from the Clinical Pharmacogenetics Implementation Consortium and Dutch Pharmacogenetics Working Group. *Clin Transl Sci.* **13**, 116-124 (2020).
